# Forging-Submerged Arc Additive Hybrid Manufacturing of the Mn-Mo-Ni Component: In Situ Reheat Cycles Inducing the Homogenization of the HAZ Microstructure

**DOI:** 10.3390/ma18010020

**Published:** 2024-12-25

**Authors:** Qiang Chi, Meijuan Hu, Jun Wang, Shuai Yan, Manye Xue, Shaojie Wu, Fangjie Cheng

**Affiliations:** 1 State Key Laboratory of Oil and Gas Equipment, CNPC Tubular Goods Research Institute, Xi’an 710077, China; chiq@cnpc.com.cn (Q.C.); wangjun1003@cnpc.com.cn (J.W.); 2 School of Materials Science and Engineering, Tianjin University, Tianjin 300350, China; ys0423@tju.edu.cn (S.Y.); xuemanye@tju.edu.cn (M.X.); shaojie@tju.edu.cn (S.W.); chfj@tju.edu.cn (F.C.); 3 Tianjin Key Laboratory of Advanced Joining Technology, Tianjin 300350, China

**Keywords:** HAZ, in situ reheat cycles, microstructure, mechanical properties

## Abstract

Forging additive hybrid manufacturing integrated the high efficiency of forging and the great flexibility of additive manufacturing, which has significant potential in the construction of reactor pressure vessels (RPVs). In the components, the heat-affected zone (HAZ, also called as bonding zone) between the forged substrate zone and the arc deposition zone was key to the final performance of the components. In this study, the Mn-Mo-Ni welding wire was deposited on the 16MnD5 substrate with a submerged arc heat source. The in situ reheat cycle effect of the submerged arc heat source on the microstructure and mechanical properties of the HAZ were studied. The results showed that the HAZ underwent four heat treatment processes, including two full austenitizing stages, one high-temperature stage, and continuous low-temperature tempering, which formed a homogenized microstructure in the HAZ and was mainly composed of tempered sorbite (Tempered-S). The HAZ microhardness is around 278.7 HV, which is about 150 HV lower than the microhardness only conducted by one thermal cycle. Furthermore, the effects of preheating the substrate and adjusting the heat inputs on the HAZ were studied. The results indicated that the clustered cementite was precipitated, which destroys the low-temperature impact toughness of the HAZ after preheating. A suitable heat input not only homogenized the microstructure within the HAZ but also promoted the transformation of grains into equiaxed grains. The −60 °C impact toughness of the HAZ was significantly increased from 96.7 J to 113 J.

## 1. Introduction

Low-alloy Mn-Mo-Ni steels, such as German 20MnMoNi55, French 16MND5, and US SA508-Gr3, which have a good combination of strength, toughness, and weldability, were commonly used in the manufacturing of a reactor pressure vessel (RPV) [1,2,3]. Low-alloy Mn-Mo-Ni steels are widely used for the main pressure boundary components of the primary circuit of the RPV and are protected by a 316 SS liner and are not exposed to the (pressurized water reactor) PWR water directly [4,5]. Nowadays, RPVs have been always welded by conventional large forgings. Nevertheless, large forgings with complex structures are difficult to manufacture successfully and are caused by errors that easily occur during the long production cycle [4]. Recently, the design concept of miniaturization has been applied in fourth-generation reactors, which have the advantages of cost effectiveness, safety, security, and proliferation resistance in reactors [6,7,8,9,10,11,12]. Hence, the method of narrow gap submerged arc welding is unsuitable for welding the connecting pipe sections of pressure vessels and the deep groove for inlet and outlet nozzles [13].

Hybrid manufacturing is an innovative process that seamlessly integrates forging and additive manufacturing [14], transcending the constraints of traditional forging in producing intricate structural components and the inefficiencies of standalone additive manufacturing in fabricating large-scale parts. It achieves an optimal balance among high productivity, low costs, and superior performance. For instance, Shen et al. [15] leveraged the technology to successfully create blades and disks featuring distinct microstructures and compositions, yielding blade-disk products with dual mechanical properties. This technological reform substantially broadened the horizons of manufacturing capabilities.

Components manufactured through forging additive hybrid manufacturing could be discernibly segmented into three distinct regions: the deposited zone, the HAZ, and the forged substrate zone [16,17,18]. Typically, the HAZ exhibited heightened sensitivity to rapid fluctuations in heat accumulation, leading to notable variations in its microstructure under the influence of multiple non-equilibrium rapid solidification processes [19]. In conventional welding processes, the HAZ was divided into the coarse-grained heat-affected zone (CGHAZ), the fine-grained heat-affected zone (FGHAZ), and the inter-critically heated heat-affected zone (ICHAZ) based on distinct thermal histories [20,21,22]. For instance, Ming et al. [23] prepared a dissimilar metal weld of SA508-52-316L using narrow gap gas tungsten arc welding. The results showed that starting from the weld center, the microstructure of the SA508 HAZ was sequential as follows: coarse martensite → fine martensite + fine bainite → bainite + a small amount of fine martensite → bainite (tempered region).

High heat input and a large width-to-thickness ratio of the submerged arc heat source exhibit unique advantages in microstructural control [24]. Li et al. [25] successfully fabricated Mn-Ni-Mo steels with a fully equiaxed microstructure using SAAM, leveraging the significant heat input from the submerged arc heat source. In their study, the components underwent multiple rapid normalizing, inter-critical annealing, and continuous tempering processes. This in situ full-thickness heat penetration characteristic offers a novel perspective for exploring the optimization or even the elimination of detrimental factors in hybrid additive manufacturing, which are caused by the degradation of mechanical properties at the joint interface induced by the CGHAZ.

In this study, the forging-submerged arc additive hybrid manufacturing technology was used to construct Mo-Mo-Ni steel hybrid components. The microstructurally homogenized HAZ was obtained in the bonding zone based on the full-thickness heat penetration characteristics of the submerged arc heat source with a high heat input. The evolution of the microstructure within the HAZ subjected to multiple thermal cycles was studied. Furthermore, the influence of the microstructure evolution of the HAZ on microhardness and low-temperature impact toughness was investigated. In addition, the effects of preheating and heat input on the microstructure of the HAZ were discussed.

## 2. Materials and Experimental Procedures

### 2.1. Forging-Submerged Arc Additive Hybrid Manufacturing Deposition

**Figure 1:**
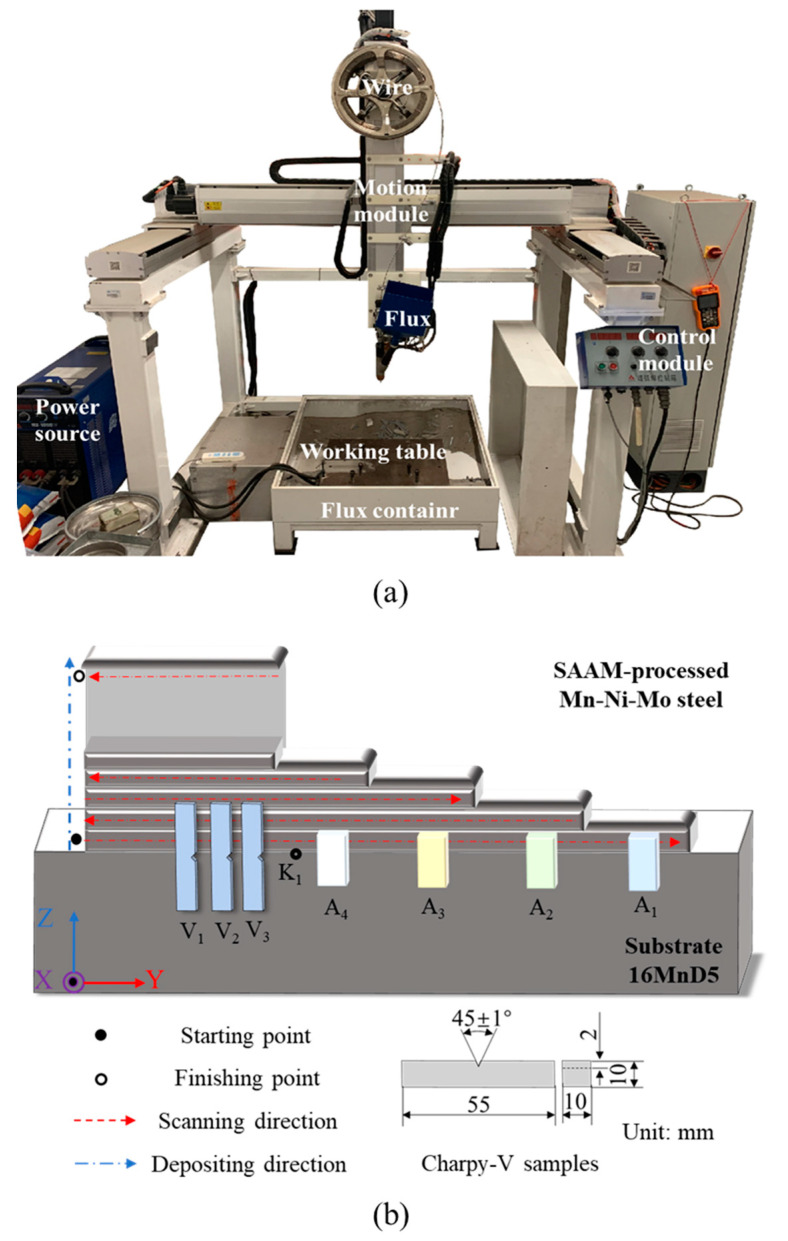
(**a**) The forging-submerged arc additive hybrid manufacturing device, (**b**) deposition strategy and representation of the extraction location of the tested samples.

The forging-submerged arc additive hybrid manufacturing equipment depicted in Figure 1a integrates a heat source (Ao-Tai MZ1000-IV Aotai, ShanDong, China), a traversing mechanism (ASDA-M), and a control system (DMCS V1.1). A nuclear power Mn-Ni-Mo type feedstock AWS 5.23 S3NiMo 1 (voestalpine Böhler Welding, Düsseldorf, Germany) with a diameter of 4.0 mm and 16MnD5 with a thickness of 60 mm were employed as the deposited material and substrate, respectively. In addition, the initial microstructure of 16MnD steel substrate is granular bainites (GBs). Additionally, to optimize the deposition process, AWS 5.23 UV 420TTR (voestalpine Böhler Welding, Düsseldorf, Germany) flux was used as the local shrouding media. The fabrication parameters utilized are detailed in Table 1. The chemical compositions of the feeding wire, substrate, and additive wall are measured by the spark direct-reading spectral analyzer, as shown in Table 2. Simultaneously, an FT-H50K handheld infrared pyrometer (KEYENCE, shanghai, China). was utilized for precise interlayer temperature monitoring, ensuring that the interlayer temperature of the components was maintained at approximately 100 °C. The K-type thermocouple was precisely welded to the designated HAZ on the substrate through manual spot welding to accurately capture the thermal history of the HAZ, as shown in Figure 1b, labeled K1. Thermodynamic calculations were conducted using JmatPro software on the nominal composition of the 16MnD5, revealing that AC_1_ is 694 °C and AC_3_ is 810 °C.

To facilitate a more precise depiction of the positional relationship between the heat source and the components, a tailored coordinate system was introduced, as illustrated in Figure 1b. The “Y” direction was designated to represent the scanning path, traversing the lengthwise dimension of the thin-walled component. The “Z” direction signified the additive path, and the “X” direction encapsulated the widthwise dimension, running parallel to the substrate plane while maintaining orthogonality to the “Y” direction.

### 2.2. Microstructural Characterization

Samples labeled A1, A2, A3, and A4 in Figure 1b were prepared to characterize the microstructural features of the HAZ within the Z-O-X plane. The samples underwent chemical etching with a 5 vol% Nital solution to enhance microstructural contrast. Subsequently, Scanning Electron Microscopy (SEM, using a JEOL-7800F, JEFO, Tokyo, Japan.) was utilized to observe the microstructures. To delve deeper into the microstructural analysis, Electron Backscatter Diffraction (EBSD) was employed. During the EBSD process, samples of 5 mm × 5 mm × 8 mm dimensions were subjected to electrochemical polishing in an electrolyte mixture comprising 90 vol% absolute ethyl alcohol and 10 vol% perchloric acid at a precisely controlled voltage of 30V for 20 s. Post-testing, the exhaustive data generated was analyzed using TSL OIM software Version7.0.

### 2.3. Mechanical Properties

According to ASTM E384, Vickers hardness measurements were performed under a load of 100 N with a dwell time of 15 s to test the microhardness of the HAZ, which had been subjected to multiple thermal cycling scenarios. In compliance with the GB/T 229-2020 standard, standard-size impact samples (10 mm × 10 mm × 55 mm) as depicted in Figure 1b were selected, with the notches positioned at the HAZ. The impact testing was conducted utilizing a high-precision ZBC2752-ED pendulum impact testing machine (MTS, Guangdong, China), and the impact samples were cooled to −60 °C. It is crucial to emphasize that each impact test result presented was a meticulously calculated average, derived from triplicate specimen testing.

## 3. Results

### 3.1. Microscopic Characterization

#### 3.1.1. Microstructure of the HAZ

**Figure 2:**
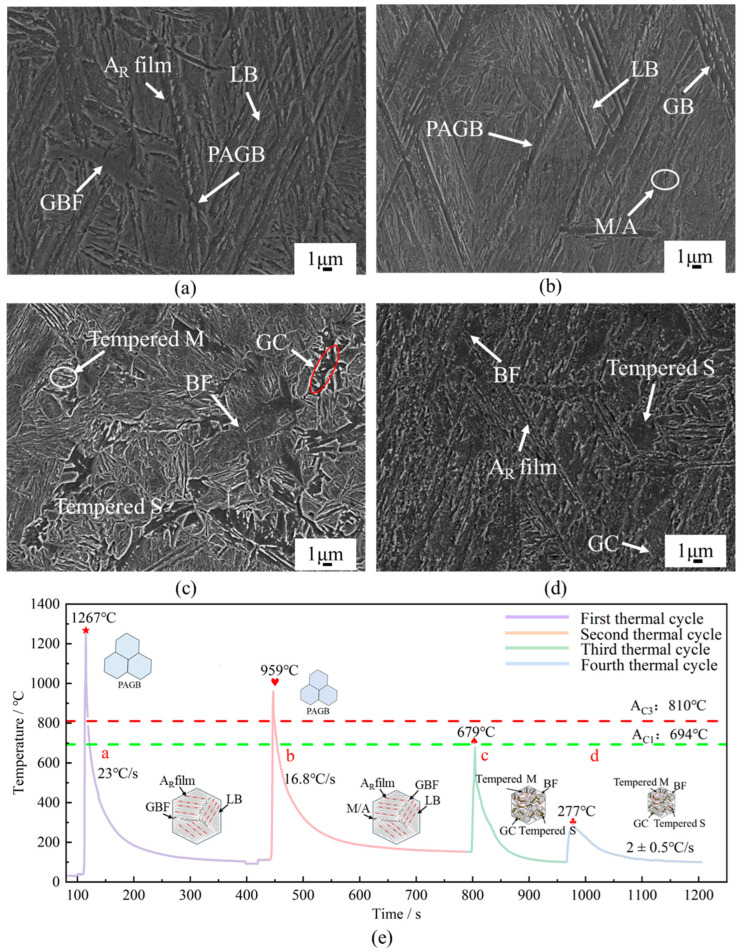
The evolution process of the microstructure of the HAZ with different thermal cycling conditions: (**a**) one thermal cycle, (**b**) two thermal cycles, (**c**) three thermal cycles, (**d**) four thermal cycles, (**e**) reheating cycle curves.

The microstructure of the HAZ under different thermal cycles is presented in Figure 2. During the first two thermal cycles, the peak temperatures of the HAZ exceeded AC_3_ (Figure 2e). After the first thermal cycle, the microstructure was completely austenitizing. The cooling rate of T_8/5_ was 23 °C/s, which provided a significant degree of supercooling and a subsequent strong driving force for the austenite-to-bainite phase transformation. Furthermore, the bainite transformation was incomplete, in which austenite was not fully consumed in some transformation regions. So, the microstructure of the HAZ was primarily composed of prior austenite grain boundaries (PAGBs), lath bainite (LB), grain boundary ferrite (GBF), and a small amount of retained austenite (A_R_), as shown in Figure 2a. After the second thermal cycle, the PAGs were refined at the rapid normalizing treatment. The cooling rate of T8/5 was 16.8 °C/s, and at this cooling rate, the proportion of LB was increased, forming bainite bundles with widths ranging from 3 to 10 μm and some granular bainites (Figure 2b). Furthermore, the precipitation of cementite between the undercooled austenite and ferrite became difficult. Influenced by the granular bainite, the remnants of undercooled austenite particles exhibited a granular morphology. At the subsequent non-equilibrium cooling process, the granular undercooled austenite with varying alloy element concentrations partially transformed into martensite. The martensite and untransformed austenite formed blocky or island-like M-A constituents at the PAGBs [26].

In the third thermal cycle, the peak temperature of the HAZ reached 679 °C (Figure 2e). This subjected the microstructure that had been formed after the second thermal cycle to high-temperature tempering. During the cooling process, the supersaturated carbon atoms within the GBF began to precipitate and segregate, leading to the formation of cementite and eventually evolving into granular cementite (GC). Concurrently, parts of ferrite underwent thermal stabilization and subsequent recrystallization, with the LB morphology gradually disappearing and transforming into polygonal ferrite (PF), as shown in Figure 2c. This mechanical mixture of PF and GC is referred to as tempered sorbite (Tempered S). Notably, the M-A constituents at GBs gradually decomposed under this condition, releasing cementite and forming bulk ferrite structures with lower carbon content. Additionally, the A_R_ films might transform into martensite or bainite, but these newly formed martensite structures soon underwent tempering again, transforming into tempered martensite (Tempered M). During the fourth thermal cycle, the peak temperature was 277 °C (Figure 2e). This subjected the microstructure that had been formed after the third thermal cycle to low-temperature tempering. The cementite of the Tempered S maintains a relatively fine morphology and is dispersed in the ferrite in a solid solution form, resulting in solid solution strengthening, as depicted in Figure 2d.

**Figure 3:**
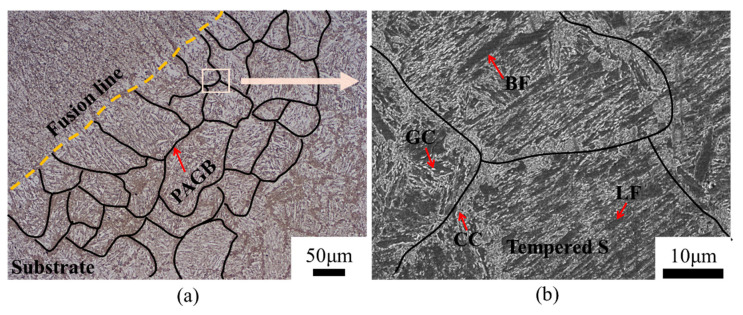
The HAZ of the components by forging-submerged arc additive hybrid manufacturing after undergoing four thermal cycles. (**a**) the microstructure of the HAZ, (**b**) a partial enlarged view of (**a**).

The heat treatment, consisting of two rapid normalizations, one high-temperature tempering, and one low-temperature tempering, was applied to the HAZ under the effect of multiple thermal cycles. Consequently, the HAZ ultimately exhibited a uniform tempered microstructure, as shown in Figure 3a. Typically, the HAZ of a weld bead is divided into the coarse-grained heat-affected zone (CGHAZ), the fine-grained heat-affected zone (FGHAZ), and the inter-critically heated heat-affected zone (ICHAZ) [20,21,22]. This division arises because it only undergoes one or two thermal cycles during the welding process. Therefore, the uniform microstructure in the HAZ is not obtainable, even using a submerged arc heat source. For example, Li et al. [27] employed narrow gap submerged arc welding to weld SA508-III, and the smooth transition region from the HAZ to base metal comprised coarse martensite → fine martensite + fine bainite → bainite.

The cementite was formed in two types, as shown in Figure 3b. One was precipitated along the PAGBs, eventually interconnecting and entangling with each other to form a complex network-like structure. The other type was precipitated within the bainitic ferrite grains, exhibiting a randomly dispersed and granular distribution. Panicker et al. [28] demonstrated that the cooling rate of the components gradually stabilized at a constant value with the number of deposited layers accumulated at the additive manufacturing process. Therefore, the subsequent additive process will maintain the low-temperature tempering state for an extended period. This allows carbon atoms to remain in a state of easy diffusion for a significant time.

#### 3.1.2. Crystallographic

**Figure 4:**
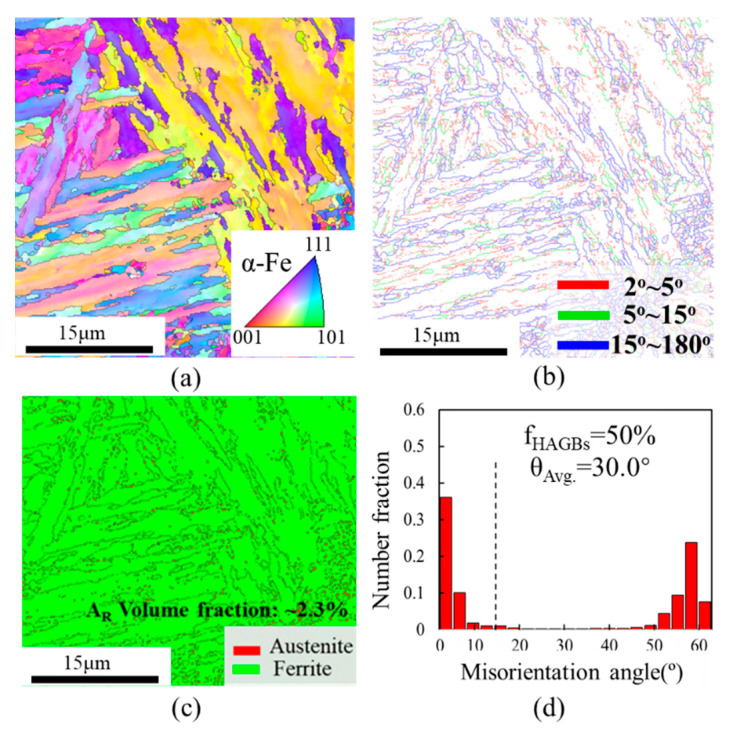
EBSD characterization of microstructures in the HAZ after undergoing four thermal cycles: (**a**) IPF map, (**b**) GB map, (**c**) phase map, (**d**) misorientation angle distribution map.

The crystallographic information of the HAZ of the sample after undergoing four thermal cycles is shown in Figure 4. The microstructure exhibited a relatively clear <001>α texture parallel to the Z-axis (Figure 4a). In fact, the various α-Fe variants present at room temperature originated from the precipitation of undercooled austenite [29,30]. Gan et al. [31] found that the orientation relationship between BF and the PAGs adhered to the classical K-S relationship. LBs nucleated and grew in the same direction and the same grain boundary (GB), exhibiting similar orientations. The lath bundles act as the actual controlling unit, and large misorientation angles were formed within the LBs [31]. Figure 4b,d showed that HAGBs were approximately 50.9%, with average misorientation angles θ_Avg_ of 30.0°. The phase map indicated that the A_R_ within the HAZ was situated within the bainite/ferrite lath boundaries, and the volume fraction of A_R_ in the HAZ was as high as 2.9% (Figure 4c). This could be attributed to the diffusion of carbon elements under the effect of non-equilibrium heat, which destroys the stability of austenite.

### 3.2. Mechanical Properties

#### 3.2.1. Microhardness

**Figure 5:**
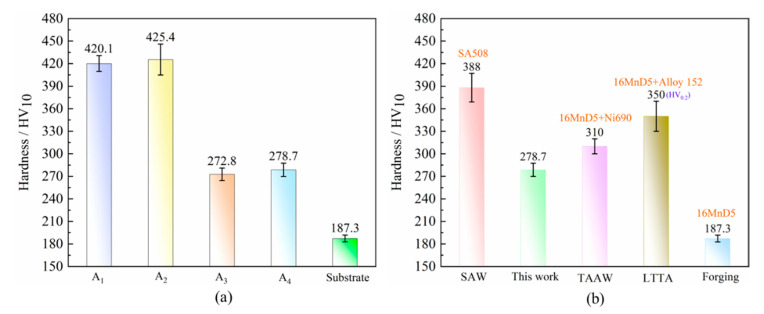
Microhardness of the HAZ after undergoing different thermal cycles: (**a**) under different thermal cycles, (**b**) under different processing techniques [32,33,34]. (SAW [32], submerged arc welding; TAAW [33], tungsten argon arc welding; LTTA [34], long-term thermal aging.)

As described above, the microstructure has been fully austenitized during the initial two thermal cycles, and the identical microstructures were formed as a result. However, the amount of hard bainite was formed due to the rapid normalizing of the second thermal cycle. The average microhardness of the HAZ after the second thermal cycle was higher than that obtained after the first thermal cycle, as shown in Figure 5a, labeled A1 and A2. After the third thermal cycle, the high-temperature tempering caused a large amount of hard bainite to transform into PF. Concurrently, the GC precipitated at the ferrite lath interfaces. The average microhardness exhibited a notable decrease, dropping to approximately 272.8 HV10 (Figure 5a, labeled A3), with a maximum of 285 HV10. However, compared to the third thermal cycle, the average microhardness increased by approximately 10 HV10 after the fourth thermal cycle. It stabilized at around 278.7 HV10, with a peak value of 298 HV10, as shown in Figure 5a, labeled A4. That was due to the solid solution strengthening, which resulted in a superior average microhardness compared to that achieved through high-temperature tempering. Additionally, Figure 5b presents a comparative assessment of the microhardness measurements of the HAZ of 16MnD5 steel, considering samples subjected to SAW [32], surfacing welding [33], thermal aging [34], and the present investigation. It was evident that the present work exhibited a relatively lower average hardness, indicating that the forging-submerged arc additive hybrid manufacturing effectively enhanced the microstructural homogenization within the HAZ.

#### 3.2.2. Charpy Impact Tests

**Figure 6:**
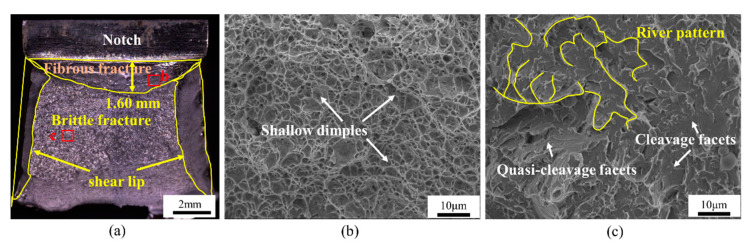
Impact fracture. (**a**). SEM of the fracture surface of the impact specimen, enlarged SEM of the selected areas marked as “b” and “c” in (**a**), (**b**). and (**c**), respectively.

The impact absorption energy of the HAZ after undergoing four thermal cycles was 96.7 ± 16.86 J, and the samples were positioned as shown in Figure 1b. In addition, the impact fracture was found to consist of shear lips, fibrous zones, and radial zones (Figure 6a). The width of the fiber zone reached approximately 1.60 mm, and the fracture surface was densely populated with numerous small, deep dimples (Figure 6b). Dimples were microscopic voids produced during the plastic deformation of the impact sample in micro-regions. The larger the number and the smaller the size of dimples, the higher the energy absorbed during deformation. In the low-temperature Charpy impact test, the presence of dimples effectively enhanced the energy required for crack propagation. The radial zone was mainly composed of cleavage planes and quasi-cleavage planes (Figure 6c).

## 4. Discussion

### 4.1. Transition of Bainites

**Figure 7:**
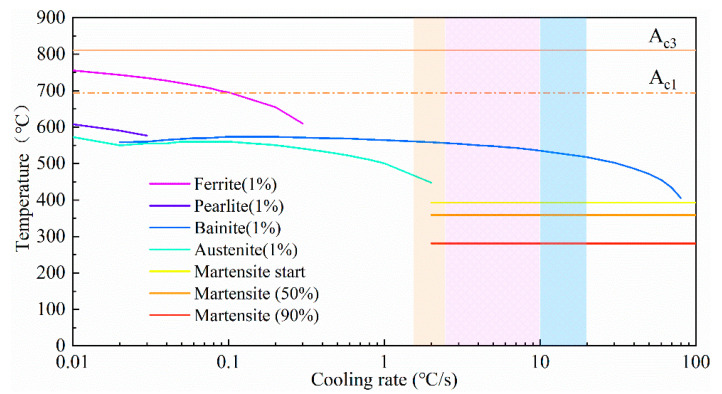
Continuous cooling transformation curve.

The CCT curve in Figure 7 indicated that, within the cooling range of 10–100 °C/s, the material system selected in this paper underwent a solid phase transition from the PAGs directly into bainite. According to the above, after the first two thermal cycles, bainite bundles distributed in different directions were formed inside the PAGs. This was because, in the early stage of the phase transformation, primary LBs nucleated at the PAGBs and grew into the grain interior. As the transformation progressed, subsequent bainite nucleated on the surface of the already formed bainite [31]. It is noteworthy that throughout the solid-state phase transformation process, a specific Kurdjumov–Sachs (K-S) orientation relationship was observed between austenite and bainite. As a result, under the impact of primary nucleation and surface-stimulated nucleation of bainite, various directions of bainite bundles, adjacent to each other, gradually formed within the PAGs. Furthermore, after the second treatment, the normalizing thermal effect on the bainite bundles initially led to an increase in the proportion of lath bainite in the microstructure. Additionally, due to the weakened heat dissipation conditions, the growth of lath structures resulted in the coarsening of bainite bundles. During the third thermal cycle, with the peak temperature falling within the high-temperature tempering range, partial bainitic ferrite underwent recovery and recrystallization, ultimately forming PF.

### 4.2. Toughening Mechanism

The toughening of metals was often closely related to both the orientation angle between adjacent fine grains and the precipitation of A_*R*_. The orientation angle between adjacent fine grains is intimately linked to the transformation of the main crack propagation path. In most cases, there was a higher probability of crack deflection occurring near the HAGB [25]. This was attributed to the fact that HAGBs usually have irregular atomic arrangements and help to form dislocation loops, which enables the GB to absorb energy more easily. During the propagation of microcracks initiated by external stress in the material, a significant amount of energy is consumed by the HAGBs. On the other hand, HAGBs usually have higher surface energy, enabling them to absorb and disperse energy effectively during material impact. Consequently, this slows down the crack propagation speed and ultimately enhances the material’s toughness [35]. In addition, the A_R_ films located at the GB positions exhibit excellent plastic deformation capabilities. These A_R_ films efficiently coordinate the deformation process, mitigate stress concentration phenomena, and subsequently enhance the toughness of the material.

**Figure 8:**
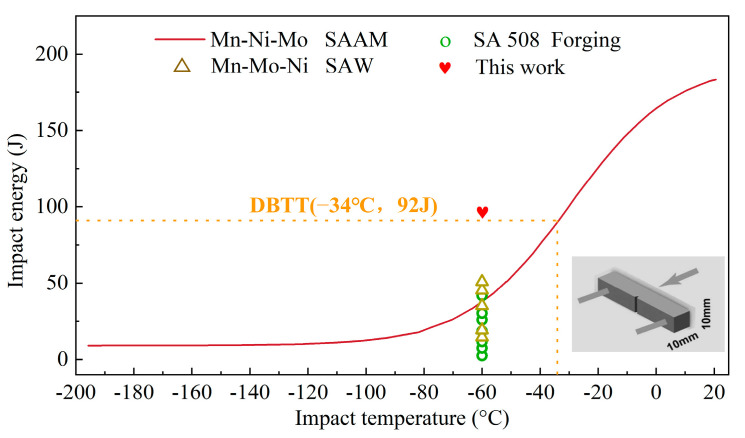
Impact toughness of the Mn-Ni-Mo alloy by SAAM, SAW, and forging [25,36,37,38,39].

Compared with other traditional processing technologies, such as SAAM, forging, and arc welding, the forging-submerged arc additive hybrid manufacturing exhibits significant advantages in low-temperature impact toughness. As evident in Figure 8, the −60 °C impact toughness of the HAZ produced by forging-submerged arc additive hybrid manufacturing has been significantly superior to that of the material used in PRV nuclear power applications, which were typically produced by conventional manufacturing methods.

### 4.3. The Effects of Preheating and Heat Input on the HAZ

The mechanism of outstanding impact toughness has been explained in detail, in Section 4.2. It can be found that the in situ reheat cycle induction plays a decisive role in improving the mechanical properties. Naturally, the processes of preheating the substrate and increasing heat input were considered to further strengthen this effect.

**Figure 9:**
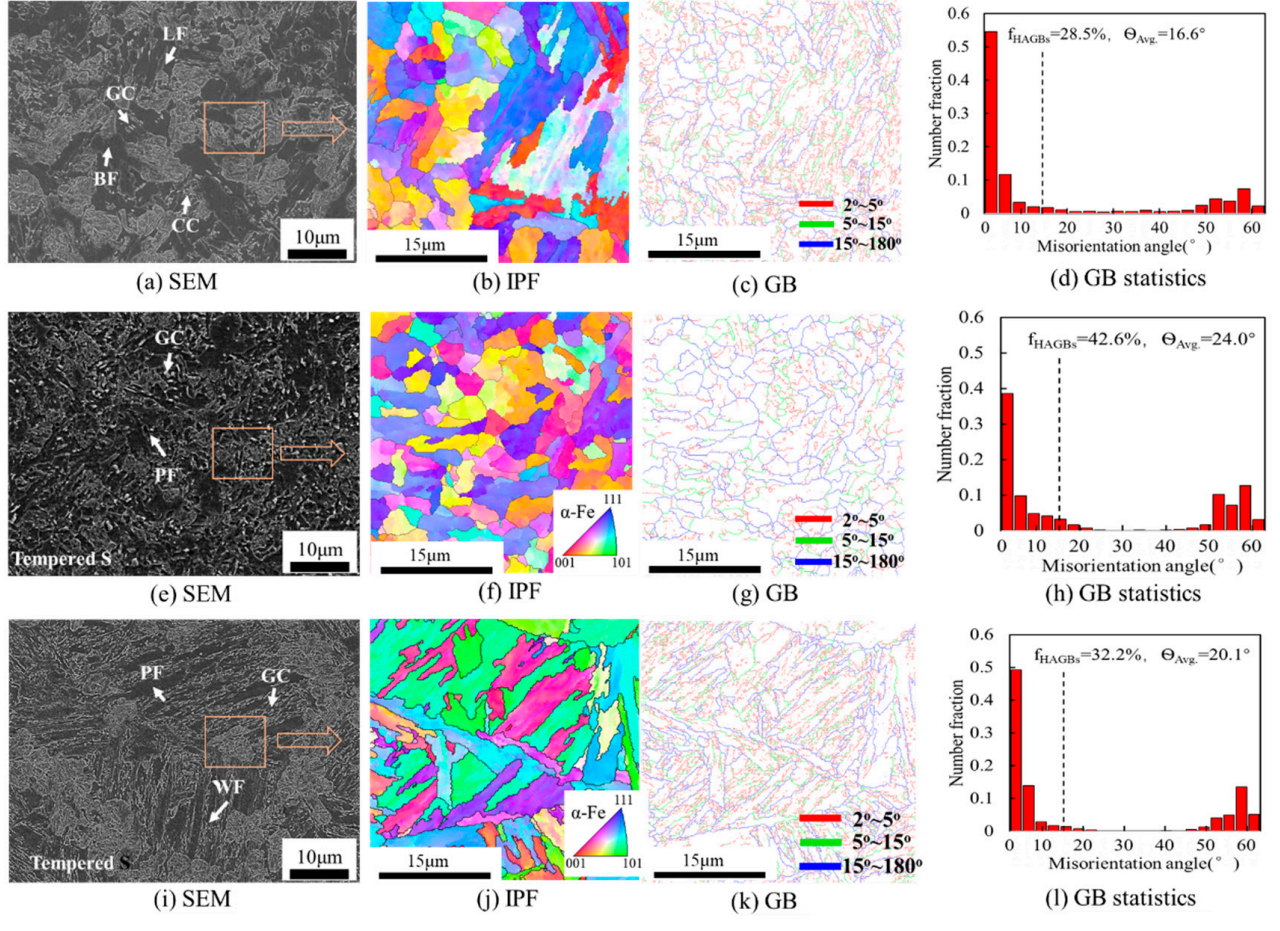
Microstructure and crystallographic characteristics of the HAZ with different heat parameters: (**a**–**d**) preheating at 150 °C of 1800 J/mm, (**e**–**h**) 2250 J/mm, (**i**–**l**) 2700 J/mm.

Firstly, the method with a substrate preheating of 150 °C and a heat input of 1800 J/mm was adopted. The carbon elements of the HAZ underwent full diffusion with ample time by preheating, which allowed the granular cementite within BF to transform gradually from a randomly dispersed distribution into a clustered arrangement. Concurrently, the morphology of ferrite shifts from lath shaped to blocky, with fine equiaxed ferrite growing larger without discernible directional growth characteristics (Figure 9a). Furthermore, the initial heat dissipation conditions deteriorate, resulting in a notable enlargement of the PAGs (Figure 9b). This reduction in the count of GBs and the presence of clustered cementite led to an increase in the proportion of LAGBs (Figure 9c,d). Consequently, its low-temperature impact absorbed energy at −60 °C was only 50 J. Therefore, the heat treatment of the substrate has not achieved the ideal result.

Subsequently, various heat inputs were explored, including 1800 J/mm, 2250 J/mm, and 2700 J/mm. The results indicated that as the heat input increased from 1800 J/mm to 2250 J/mm, the morphology of cementite in the HAZ transformed from a coexistence of network and granular forms to primarily granular. Concurrently, the microstructure evolved from a coexistence of lath ferrite and fine equiaxed ferrite to one dominated by equiaxed ferrite. Notably, both the proportion of HAGBs and the average GB angles declined slightly. Consequently, the low-temperature impact toughness increased from 96.7 J to 113 J. However, the low-temperature impact toughness significantly declined at a heat input of 2700 J/mm. This is due to ferrite nucleating at the PAGBs and growing into the interior of PAGs (Figure 9j), resulting in the formation of an overheated defect structure known as Widmanstätten ferrite (Figure 9j). Meanwhile, this overheated defect structure had a lower carbon content, and the supersaturated carbon elements diffused into the surroundings. Consequently, the carbon balance within the PAGs was disrupted, leading to the precipitation of network-like cementite along the PAGBs once again (Figure 9e,i). Additionally, the proportion of HAGBs and the average GB angles were only 32.2% and 20.1°, respectively, as shown in Figure 9j–l.

## 5. Conclusions

This paper investigated the microstructural evolution and the relationship between the microstructure and properties in the HAZ, which were fabricated by the forging-submerged arc additive hybrid manufacturing under multiple thermal cycles. The following main conclusions were drawn.

1. A hybrid Mn-Mo-Ni component with the matrix retained was successfully obtained through forging-submerged arc additive hybrid manufacturing. The microstructure of the HAZ evolved from heterogeneous in various regions to homogeneous throughout by the in situ reheat cycles of the submerged arc heat source, without the presence of sub-zones.
2. The HAZ was mainly composed of cementite and PF. The presence of PF promoted the formation of more than 50% of HAGBs. The microhardness of the HAZ was approximately 278.7 HV10, and the impact absorption energy at −60 °C was 96.7 ± 16.8 J, under the unique in situ reheating cycle of the submerged arc heat source.
3. The appropriate heat input can better exploit the in situ reheat cycles of the submerged arc heat source to induce the formation of fully equiaxed grains within the HAZ, which enhances the low-temperature impact toughness. In this paper, an appropriate heat input resulted in an increase in low-temperature impact toughness from 96.7 J to 113 J at −60 °C.

## Figures and Tables

**Table 1:** Optimized parameters used in the forging-submerged arc additive hybrid manufacturing process.

Voltage (V)	Current (A)	Travel Speed (mm/s)	Heat Input (J/mm)	Contact Tip-Substrate Distance (mm)	Interlayer Temperature (℃)	Wire Feed Rate (mm/s)
27	400	6	1800	28	100	DCEP
27	500	6	2250	28	100	DCEP
27	600	6	2700	28	100	DCEP

**Table 2:** Nominal chemical compositions of the feedstock, substrate, and the forging-submerged arc additive hybrid manufacturing processed component (wt%).

	C	Mn	Si	S	P	Cr	Ni	Mo	Fe
Feedstock S3NiMo1	0.10	1.72	0.24	0.002	0.009	0.01	0.97	0.54	Bal.
Substrate 16MnD5	0.15	1.35	0.20	0.005	0.008	0.13	0.65	0.50	Bal.
Additive wall	0.06	1.89	0.36	0.001	0.011	0.03	0.93	0.54	Bal.

## Data Availability

Data is contained within the article.
